# Rapid and Specific Detection of All Known *Nipah virus* Strains’ Sequences With Reverse Transcription-Loop-Mediated Isothermal Amplification

**DOI:** 10.3389/fmicb.2019.00418

**Published:** 2019-03-11

**Authors:** Liping Ma, Zhen Chen, Wuxiang Guan, Quanjiao Chen, Di Liu

**Affiliations:** ^1^CAS Key Laboratory of Special Pathogens and Biosafety, Wuhan Institute of Virology, Chinese Academy of Sciences, Wuhan, China; ^2^Computational Virology Group, Wuhan Institute of Virology, Chinese Academy of Sciences, Wuhan, China; ^3^University of Chinese Academy of Sciences, Beijing, China; ^4^Center for Emerging Infectious Diseases, Wuhan Institute of Virology, Chinese Academy of Sciences, Wuhan, China

**Keywords:** *Nipah virus*, reverse transcription-loop-mediated isothermal amplification, RT-LAMP, rapid detection, N gene

## Abstract

*Nipah virus* (NiV) is a zoonotic virus and can be transmitted through contaminated food or directly between people. NiV is classified as a Biosafety Level 4 agent, not only because of its relatively high case fatality rate, but also because there is no vaccine or other medical countermeasures and it appears to be transmitted by fomites/particulates. The development of rapid detection assay for NiV is of great importance because no effective field test is currently available. In this study, an isothermal (65°C) reverse transcription-loop-mediated isothermal amplification (RT-LAMP) method was developed, targeting the nucleocapsid protein (N) gene, for the rapid detection of NiV, and was compared with conventional RT-PCR. Three pseudoviruses of NiV N gene representing all known strains were constructed to replace live NiV. A set of RT-LAMP primers, targeting a highly conserved region of the N gene in the viral genome was designed to identify all known NiV strains. Sensitivity tests indicated that the detection limit of the RT-LAMP assay was approximately 100 pg of total NiV pseudovirus RNA, which is at least 10-fold higher than that of conventional RT-PCR. Specificity tests showed that there was no cross-reactivity with nucleocapsid protein gene of *Hendra virus*, *Newcastle disease virus*, *Japanese encephalitis virus*, or *Influenza A virus*. The RT-LAMP assay provides results within 45 min, and requires no sophisticated instruments, except an isothermal water bath or metal bath with 1 μl calcein indicator. An analysis of the clinical samples showed that the assay had good stability. In conclusion, systematic experiments have shown that the RT-LAMP assay developed here effectively detects three NiV pseudoviruses representing all known strains of NiV, with high specificity, sensitivity and stability.

## Introduction

*Nipah virus* (NiV) belongs to the family *Paramyxoviridae* and genus *Henipavirus*, which contains another four species: *Hendra virus* (HeV), *Cedar virus*, *Mojiang virus*, and *Kumasi virus* (McLinton et al., 2017). NiV is classified as a Biosafety Level 4 (BSL-4) agent because of its high pathogenicity and mortality rate, which ranges from 40 to 100% (SEARO, 2014), and causes fatal human encephalitis and respiratory disease when it spills over from its bat reservoir – *Pteropod* fruit bats (flying foxes). It can also infect pigs and horses. NiV infection was first described in Malaysia and Singapore in 1998–1999 after a massive outbreak of fatal encephalitis among humans (283 cases, 109 deaths) (Chua et al., 2000). Outbreaks of fatal encephalitis and respiratory disease attributable to NiV have been reported in Bangladesh and India since 2001 (Chadha et al., 2006; Luby et al., 2009; Arankalle et al., 2011; Lo et al., 2012).

*Nipah virus* is enveloped by a filamentous nucleocapsids (Halpin et al., 2011), and has a single-stranded negative-sense RNA genome of 18,246 nt (Malaysian isolate) or 18,252 nt (Bangladeshi isolate) (Harcourt et al., 2005), which consists of six genes encoding six major structural proteins: nucleocapsids (N), phosphoprotein (P), matrix protein (M), fusion protein (F), glycoprotein (G), and large protein (L) or RNA polymerase. The first 12 nucleotides of the 5′ and 3′ genomic termini are highly conserved and complementary (Wang et al., 2001). There are two typical genotypes of NiV: genotype M (containing the Malaysian isolates, Cambodian isolates, and some of Thai bat RNAs) and genotype B (containing Bangladeshi isolates, Indian isolates, and other Thai bat RNAs) (Lo et al., 2012; Wacharapluesadee et al., 2016), and the similarity between the genomes of the Bangladeshi and Malaysian isolates is 91.8% (Harcourt et al., 2005).

Loop-mediated isothermal amplification (LAMP) was first developed in 2000 by Notomi et al. (2000), and can amplify DNA effectively and with high specificity under isothermal conditions. DNA is synthesized by the *Bacillus stearothermophilus* (*Bst*) DNA polymerase large fragment, which has high chain- displacement activity. The reaction is performed under isothermal conditions ranging from 60 to 65°C, so no time is lost in thermal transition. Because a set of specific primers can identify at least six different fragments, the reaction is highly specific to the target sequence. The addition of loop primers improves the LAMP reaction (Nagamine et al., 2002), reducing the reaction time and potentially increasing its sensitivity. Positive samples can be detected qualitatively and visualized as increased turbidity, or quantified by measuring the turbidity of the reaction. Furthermore, the addition of a calcein indicator allows positive results to be read with the naked eye (Tomita et al., 2008) based on a color change. Therefore, compared with other nucleic acid amplification methods, LAMP assay has the advantages of specificity and rapidity. In recent years, the technology has been widely used in the detection of pathogen, especially viruses, both in China and abroad, including in the families *Orthomyxoviridae* (Imai et al., 2007), *Hepadnaviridae* (Zhao, 2017), *Flaviviridae* (Kwallah et al., 2013; Deng et al., 2015; Hu et al., 2015; Cao et al., 2016; Zhao, 2017), *Togaviridae* (Parida et al., 2007), *Rhabdoviridae* (Soliman and Ei-Matbouli, 2006), *Herpesviridae* (Iwata et al., 2006; Wang et al., 2015), *Arenaviridae* (Fukuma et al., 2011), *Filoviridae* (Kurosaki et al., 2010, 2016), *Paramyxoviridae* (Foord et al., 2012), and *Picornaviridae* (Lei et al., 2014).

In this study, we developed an reverse transcription-loop-mediated isothermal amplification (RT-LAMP) assay to detect NiV. Our purpose was to determine whether the assay recognizes the conserved region of the NiV genome and can therefore be used to detect all known strains of NiV. The specificity, sensitivity and stability of the RT-LAMP assay were investigated to confirm whether it offers a supplementary method for the molecular identification of NiV.

## Materials and Methods

### Pseudoviruses, Plasmids and Viral RNA

Thirty-five complete NiV N genes (accession numbers listed in Supplementary Table S1) were aligned using BioEdit sequence Alignment Editor version 7.1.3.0 to identify candidate N genes to represent all known NiV strains. Analysis of the multiple sequence alignment identified three complete N genes (1599 bp) of NiV that typically represented three types of NiV: Malaysian isolate (GenBank accession KY425646.1), Bangladeshi isolate (GenBank accession JN808862.1), and N gene sequence from Thai bat RNAs (GenBank accession KT163255.1). These were synthesized by Tsingke Biological Technology Co., Ltd. (Wuhan, China), and inserted into pUC57 vector (Tsingke Biological Technology Co., Ltd., Wuhan, China), generating pUC57-NiV-MN, pUC57-NiV-BN, and pUC57-NiV-TN, respectively. In subsequent experiments, these three plasmids were used to optimize the LAMP reaction system and as positive control.

We then conducted the pseudoviruses of the three NiV N genes by inserting the three N genes into the pHAGE-CMV-MCS-IZsGreen vector (7742 bp) provided by Center for Emerging Infectious Diseases, Wuhan Institute of Virology (Chinese Academy of Sciences, Wuhan, Hubei), generating pha-NiV-MN, pha-NiV-BN, and pha-NiV-TN, respectively. The 293T cell line was transfected individually with each construct. Fluorescence was observed after 48 h and the cells were collected. The supernatant was collected after three rounds of freeze-thaw in a liquid nitrogen and a 37°C water bath for 5 min each.

The viral RNA was extracted by a Nucleic Acid Extraction System with matched EX-RNA/DNA viral nucleic acid extraction kits (Tianlong Science and Technology, Co., Ltd.). The genomes of *Japanese encephalitis virus* vaccine strain SA14-14-2 (JEV, provided by China Virus Resource and Information Center, Wuhan, China), the A/Puerto Rico/8/1934(H1N1) (PR8) strain of *Influenza A virus* (maintained in our lab) and the related paramyxovirus *Newcastle diseases virus* (NDV, isolated and maintained in our laboratory), were extracted, as well as the RNA of pHAGE-CMV-MCS-IZsGreen vector were extracted. Because live HeV was unavailable, the complete N gene (1599 bp) of HeV (GenBank accession HM044318.1) was synthesized by Tsingke Biological Technology Co., Ltd., and inserted into pET-28a^+^ vector (Tsingke Biological Technology Co., Ltd., Wuhan, China) (pET-HeV) to be used instead of live HeV. The RNA of HeV N gene was then generated by HiScribe^TM^ T7 Quick High Yield RNA Synthesis Kit (New England Biolabs Inc., Ipswich, MA, United States) according to the standard RNA synthesis protocol.

### Primer Design

The NiV N gene is highly conserved and is often used for Reverse Transcription-Polymerase Chain Reaction (RT-PCR) detection of NiV (Guillaume et al., 2004). Therefore, a total of 35 complete sequences of NiV N gene (1599 bp) was downloaded from GenBank and aligned to identify the conserved region. Three sets of primers containing degenerate bases were designed to detect all known NiV strains, using the PrimerExplorer 5.0 software^1^, as the candidate primers. All the primers were synthesized by Tsingke Biological Technology Co., Ltd.

### RT-LAMP Assay

The RT-LAMP reaction was performed in a 25.0 μl volume, containing 0.5 mM betaine (5 mM stock, Sigma-Aldrich Inc., St Louis, MO, United States), 1.4 mM each dNTP (10.0 mM stock, TaKaRa Bio Inc., Clontech Laboratories, Inc., Dalian, China), 2.5 μl of 10× ThermoPol Reaction Buffer [(1 × ThermoPol Reaction Buffer contains 20.0 mM Tris–HCl, pH 8.8; 10.0 mM KCl; 2.0 mM MgSO_4_; 10.0 mM (NH_4_)_2_SO_4_; 0.1% Triton X-100) New England Biolabs Inc.], 6.0 mM MgSO_4_ (New England Biolabs Inc.), 32 pM each of forward inner primer (FIP) and backward inner primer (BIP), 15 pM each of forward loop (LF) and backward loop (LB) primers (or replaced with an equal volume of nuclease-free water), 4 pM each of forward outer (F3) and backward outer (B3) primers, 8.0 U of *Bst* DNA polymerase large fragment (New England Biolabs Inc.), 7.5 U of WarmStart RTx reverse transcriptase (New England Biolabs Inc.; only added to the reaction system when the template was RNA), and 2.0 μl of pseudovirus RNA (≥50 pg/μl). The RNA from the cell extract transfected with pHAGE-CMV-MCS-IZsGreen vector and nuclease-free water were used as the negative controls, and the plasmids containing the three N genes were used as the positive controls (mixed without 7.5 U of WarmStart RTx reverse transcriptase). The mixture was covered with a grain of sealant (low-melting-point wax, melting point: 48.0–50.0°C), and incubated for 60 min in a Realtime Turbidimeter LA-320C (Eiken Chemical Co., Ltd., Tochigi, Japan) to collect the turbidity data at 61–65°C, followed by incubation at 75°C for 5 min to inactivate the enzymes. An increase in turbidity of >0.1 and a peak time greater than the negative controls indicated a positive sample. Calcein indicator (1 μl, Eiken Chemical Co., Ltd., Tochigi, Japan) was added to monitor the RT-LAMP reaction with naked eye, and the reaction was incubated in a water bath at 61–65°C for 50 min, and then heated at 80°C for 5 min to inactivate the enzymes. The results were determined according to the color change, with fluorescent green indicating positive samples and orange indicating negative samples. All the experiments were performed in triplicate.

### Conventional RT-PCR

Conventional RT-PCR was conducted to compare its detection sensitivity with that of the RT-LAMP assay. The pseudoviruses RNA and the RNA from the cell extract transfected with pHAGE-CMV-MCS-IZsGreen vector were reverse transcribed with a random primer, and then 10-fold diluted to 10^−6^ with nuclease-free water. The plasmids containing the three NiV N genes, pUC57-NiV-BN, pUC57-NiV-MN, and pUC57-NiV-TN, were used as the positive controls, and the cDNA of pHAGE-CMV-MCS-IZsGreen vector and nuclease-free water were used as the negative controls. The specific primers of RT-PCR targeted the N gene of NiV and their sequences were: NiV-N-F 5′-GGAGTTATCAATCTAAGTTAG-3′, 5′-NiV-N-R CATAGAGATGAGTGTAAAAGC-3′ (Chadha et al., 2016). The length of the product was 159 bp. The results were visualized with 1.2% agarose gel electrophoresis.

### Clinical Sample Analysis

Clinical samples contain various impurities, which usually reduce the sensitivity of a PCR reaction. To determine the sensitivity of the RT-LAMP assay in clinical samples, an experiment was performed using four common clinical samples: blood sample, fecal sample, throat swab sample, and urine sample. The four common clinical samples were collected from the same normal healthy person and the genomes were extracted from them. The three NiV pseudoviruses RNA were then diluted 10-fold with each clinical sample genomic solution to the same concentration with the 10-fold gradient dilution of pseudoviral RNA templates, respectively, and used as the templates. The pseudoviruses RNAs were used as positive controls, and the RNA from the cell extract transfected with pHAGE-CMV-MCS-IZsGreen vector and the four samples genome solution were used as negative controls.

## Results

### Pseudovirus Concentrations

To test the specificity and sensitivity of the RT-LAMP assay against NiV genotype M and genotype B, we constructed three pseudoviruses representing all the known strains of NiV by transfecting the 293T cell line with pha-NiV-MN, pha-NiV-BN, or pha-NiV-TN. After fluorescence was observed, the 293T cells infected with the NiV pseudoviruses were collected. The RNA of the pseudoviruses was extracted and the concentrations were determined by a Qubit 3.0 Fluorometer (Life Invitrogen, United States) with the Qubit^TM^ RNA BR (Road Rang) Assay Kit (Life Invitrogen). The concentrations of the three pseudoviruses were 68 ng/μl (pha-NiV-BN), 75 ng/μl (pha-NiV-MN), and 88.6 ng/μl (pha-NiV-TN), respectively.

### Optimal RT-LAMP Primers and Conditions

**FIGURE 1:**
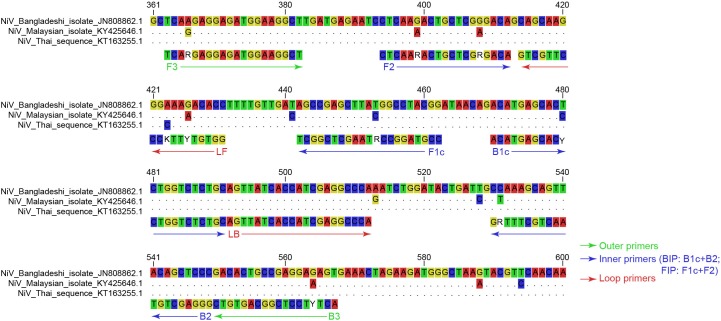
Sequence alignment of the conserved region of NiV N gene targeted for RT-LAMP detection. The target regions of each representative genotype strain were aligned. Nucleotides identical to those of NiV Bangladeshi isolate (GenBank accession JN808862.1) are replaced with dots. The primer sequences are shown below the aligned sequence, and the positions are numbered based on the complete N gene of Bangladeshi isolate (GenBank accession JN808862.1). Outer primers, inner primers (BIP and FIP primers are defined), and loop primers are shown with green arrows, blue arrows, and red arrows, respectively. The colors of the four bases differ, and the nine degenerate bases are not colored.

**FIGURE 2:**
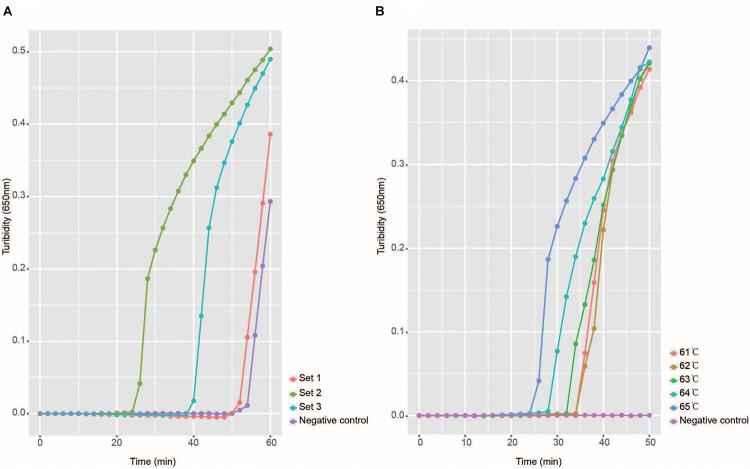
Selection of optimal reaction conditions. Panel **A** shows the turbidity signals of the three candidate primer sets, which indicate that the set two primers were optimal. This set of primers was used in all later studies. Panel **B** shows the temperature selection results, and the turbidity at each temperature is shown in a different color.

To identify the conserved region of NiV N gene, 35 complete N genes of NiV were aligned. An analysis of the alignment identified 300 bp of the N gene at positions from 301 to 600 (N gene numbering) which were selected as the conserved region and used to design the RT-LAMP primers. Three sets of primers were tested as the candidate primers. The three sets of primers were then tested with the pUC57-NiV-BN plasmid to establish their efficacy in the LAMP detection of NiV. Finally, one set of primers, which recognized eight distinct regions of 205 bp in the conserved region of the N gene (Figure 1), and consisted of six primers (two outer primers F3 and B3, two inner primers FIP and BIP, and two loop primers LF and LB) containing nine degenerate bases (Table 1), most efficiently and rapidly detected the NiV N gene (Figure 2A).

**Table 1:** RT-LAMP primers for detection of all known NiV strains based on the conserved region of the N gene.

Primer	Position^a^	Sequence (5′–3′)^b^
Forward outer (F3)	363–382	TCARGAGGAGATGGAAGGCT
Backward outer (B3)	550–567	ACTYTCCTCGGCAGTGTC
Forward inner (FIP: F1c+TTTT+F2)	442–462, 394–412	CCGTAGGCCRTAAGCTCGGCT**TTTT**CTC AARACTGCTCGRGACA
Backward inner (BIP: B1c+TTTT+B2)	470–491, 530–549	ACATGAGCACYCTGGTCTCTGC**TTTT**GG GAGCTGTAACTGCTTTRG
Forward loop (LF)	413–431	GGTGTYTTKCCCTTGCTGC
Backward loop (LB)	492–512	AGTTATCACCATCGAGGCCCA

*^a^Position numbers are based on the complete N gene of NiV Malaysian strain (GenBank No. KY425646.1), Bangladeshi strain (GenBank No. JN808862.1), and N gene sequence from Thai bat RNAs (GenBank No. KT163255.1). ^b^Bold face indicates the TTTT spacer between F1c and F2 or B1c and B2.*

To determine the best reaction temperature for the detection of NiV, five temperatures (61–65°C) were tested with the pUC57-NiV-BN plasmid. After comparison of the turbidity results, 65°C was considered the best reaction temperature (Figure 2B). The most suitable detection time with the Realtime Turbidimeter was 45 min because the negative control peak may appear as a false positive after 45 min.

### Specificity and Detection Limit of the RT-LAMP Assay

**FIGURE 3:**
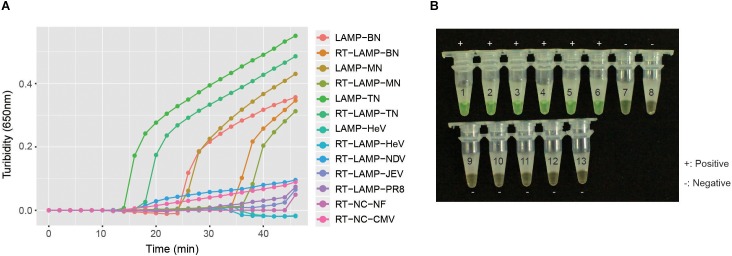
The specificity of the RT-LAMP assay. Panel **A** shows the turbidity results of the specificity test of NiV. The positive controls were the three NiV N gene plasmids pUC57-NiV-BN (Bangladeshi isolate), pUC57-NiV-MN (Malaysian isolate), and pUC57-NiV-TN (N gene sequence from Thai bat RNAs), marked as LAMP-BN, LAMP MN, and LAMP-TN, respectively. LAMP detection of pET-HeV is marked as LAMP-HeV. RT-LAMP detections of the three pseudoviruses (pha-NiV-BN, pha-NiV-MN, and pha-NiV-TN) and the RNA of pET-HeV, *Newcastle Disease virus* (NDV), *Japanese encephalitis virus* vaccine strain SA14-14-2 (JEV), and *Influenza A virus* A/Puerto Rico/8(H1N1) (PR8) are labeled RT-LAMP-BN, RT-LAMP-MN, RT-LAMP-TN, RT-LAMP-HeV, RT-LAMP-NDV, RT-LAMP-JEV, and RT-LAMP-PR8, respectively. Negative controls were the RNA from the cell extract transfected with pHAGE-CMV-MCS-IZsGreen vector and nuclease-free water, and are marked as RT-NC-CMV and RT-NC-NF, respectively. Panel **B** shows the specificity of the assay, using detection with 1 μl of calcein indicator. Numbers 1–13 represent LAMP-BN, LAMP-MN, LAMP-TN, RT-LAMP-BN, RT-LAMP-MN, RT-LAMP-TN, LAMP-HeV, RT-LAMP-HeV, RT-LAMP-NDV, RT-LAMP-JEV, RT-LAMP-PR8, RT-NC-CMV, and RT-NC-NF, respectively. Positive samples are marked “+” and negative samples are marked “−.”

To investigate the specificity of the RT-LAMP primers in detecting NiV, the RNA of pha-NiV-BN, pha-NiV-MN, pha-NiV-TN, JEV, *Influenza A virus* PR8 strain, the related paramyxovirus pET-HeV, pET-HeV RNA and NDV, the pHAGE-CMV-MCS-IZsGreen vector, and the nuclease-free water were detected for verification. The results showed that the RT-LAMP primers were specific for NiV N gene of the two genotypes, and only detected pha-NiV-MN, pha-NiV-BN, and pha-NiV-TN. No cross-reaction with JEV, *Influenza A virus* PR8 strain, the related paramyxovirus pET-HeV, pET-HeV RNA and NDV, the pHAGE-CMV-MCS-IZsGreen vector, or nuclease-free water was detected. The results are shown in Figure 3.

**FIGURE 4:**
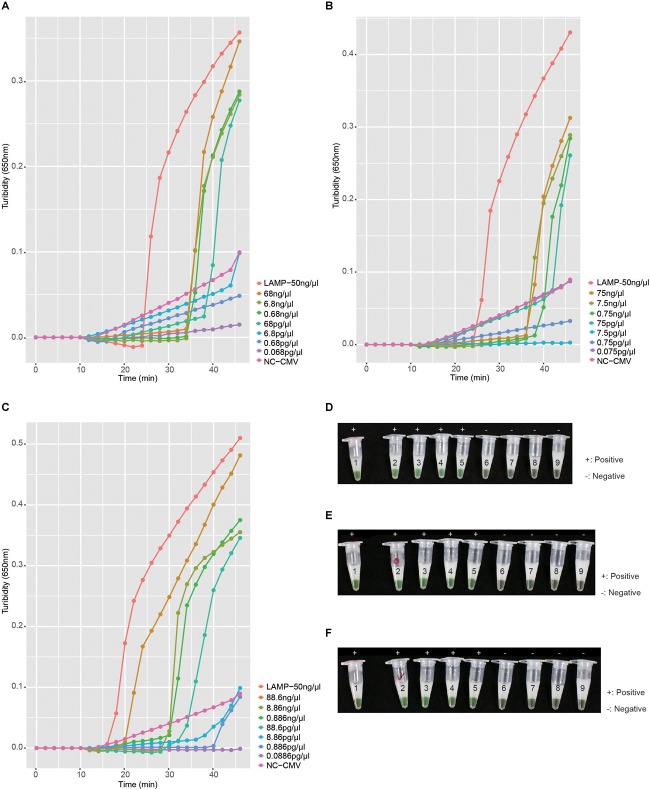
Sensitivity of the RT-LAMP assay. Panels **A–C** show the turbidity in the RT-LAMP sensitivity tests for pha-NiV-BN, pha-NiV-MN, and pha-NiV-TN, respectively. LAMP-50 ng/μl of each panel represents the positive controls: pUC57-NiV-BN (50 ng/μl), pUC57-NiV-MN (50 ng/μl), and pUC57-NiV-TN (50 ng/μl), respectively. Negative control (RNA from the cell extract transfected with pHAGE-CMV-MCS-IZsGreen vector) in each panel is marked with NC-CMV and the concentrations tested in each RT-LAMP reaction are listed on the right of each panel. Panels **D–F** show the chromogenic test results for the RT-LAMP detection of pha-NiV-BN, pha-NiV-MN, and pha-NiV-TN, respectively, with 1 μl of calcein indicator. Numbers on the tubes of each panel correspond to the legends in panels **A–C**, respectively. Positive results are marked “+” and negative results are marked “−.”

To investigate the detection limit of the RT-LAMP assay, the RNA of pha-NiV-BN, pha-NiV-MN, and pha-NiV-TN was diluted 10-fold to 10^−6^ with nuclease-free water. The assay can detect NiV pseudovirus RNA to a dilution of 10^−3^ equivalent to approximately 100 pg of the total pseudovirus RNA. There was 100% agreement between the results obtained with turbidimetry and with visual inspection with 1 μl of calcein indicator. The sensitivity analysis of the RT-LAMP assay is shown in Figure 4.

### Detection Limit of Conventional RT-PCR

**FIGURE 5:**
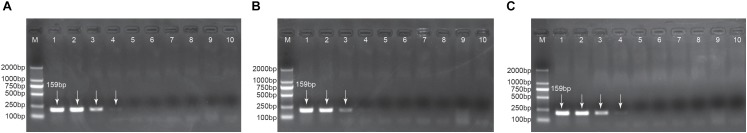
Detection limits of conventional RT-PCR. Panels **A–C** show the RT-PCR detection limits of pha-NiV-BN, pha-NiV-MN, and pha-NiV-TN, respectively. Lane M: DL2000 DNA Marker was used as the size marker; lane 1: positive controls, pUC57-NiV-BN, pUC57-NiV-MN, and pUC57-NiV-TN in panels **A–C**, respectively; lanes 2–6: 10^−1^–10^−6^ dilutions of the cDNA of pha-NiV-BN, pha-NiV-MN, and pha-NiV-TN, respectively; lanes 8–9 in the three panels: cDNA from the cell extract transfected with pHAGE-CMV-MCS-IZsGreen vector and nuclease-free water, respectively (negative controls). The lengths of products are labeled in lane 1 of each panel. Positive results are indicated with white arrows.

To compare the sensitivity of the RT-LAMP assay in detecting NiV and that of conventional RT-PCR, the 10-fold diluted NiV pseudoviruses RNAs were used as templates of the conventional RT-PCR. The RT-PCR results of each NiV pseudovirus were visualized with 1.2% agarose gel electrophoresis. The detection limit of conventional RT-PCR was 10^−2^ dilution for pha-NiV-BN and pha-NiV-TN, equivalent to approximately 1000 pg (1 ng) and 10^−1^ dilution for pha-NiV-MN, equivalent to approximately 10,000 pg (10 ng). The sensitivity of RT-LAMP detection was 10^−3^ dilution, equivalent to approximately 100 pg, indicating that the sensitivity of RT-LAMP assay was at least 10-fold higher than that of conventional RT-PCR. The results of conventional RT-PCR are presented in Figure 5.

### Stability of the RT-LAMP Assay

**FIGURE 6:**
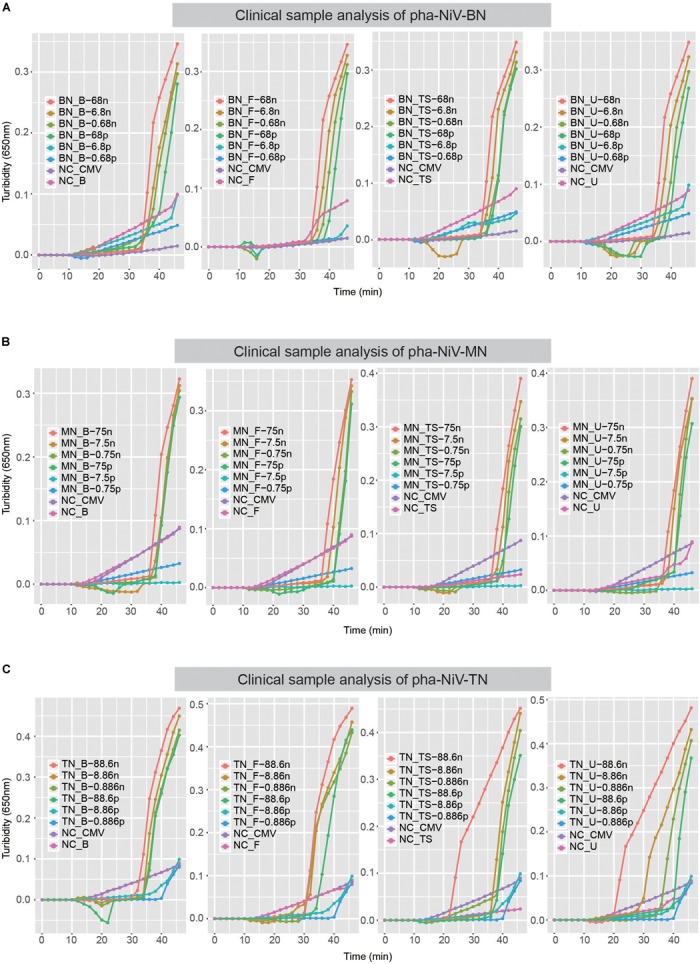
Stability of the RT-LAMP assay in clinical samples. Panel **A–C** show the turbidity-based RT-LAMP detection limits for pha-NiV-BN, pha-NiV-MN, and pha-NiV-TN, respectively, in clinical samples. The four diagrams, from left to right in each panel, represent the blood clinical sample, fecal clinical sample, throat swab clinical sample, and urine sample, which are abbreviated “B,” “F,” “TS,” and “U,” respectively. “n” and “p” in panels **A–C** represent “ng/μl” and “pg/μl,” respectively. The negative controls (RNA from the cell extract transfected with pHAGE-CMV-MCS-IZsGreen vector and the genomic solutions extracted from four common clinical samples taken of the same normal person) in each panel are marked NC-CMV, NC-B, NC-F, NC-TS, and NC-U, respectively.

To determine the stability of the RT-LAMP assay in detecting NiV in clinical samples, four clinical sample from the same normal healthy person were tested: blood sample, fecal sample, throat swab sample, and urine sample. After verification, the sensitivity of this assay in detecting NiV in clinical samples was the same as that observed in the sensitivity tests, 10^−3^ dilution, equivalent to approximately 100 pg. Therefore, the RT-LAMP assay developed here to detect NiV is effective in clinical samples. The results are shown in Figure 6.

## Discussion

*Nipah virus*, a BSL-4 pathogen with high mortality rate, presents an extreme threat to public health. Recently, the World Health Organization (WHO) summarized that up to May 31, 2018, 15 people in south India had tested positive for NiV, 13 of whom had died. Before then, on May 19, 2018, in Kozhikode District, Kerala State, India, four deaths were attributed to NiV infection, three in a single family and the fourth in a healthcare worker treating the family. Acute respiratory distress syndrome and encephalitis were observed (WHO, 2018). Notably, this is the third NiV outbreak in India, with two others reported in 2001 and 2007, respectively (Clayton, 2017). Therefore, an effective identification assay for NiV is required to establish prevention and control measures for this virus.

The laboratory tests for NiV are usually based on cell culture, virus isolation, a serological assay, such as an enzyme-linked immunosorbent assay (ELISA), immunohistochemistry, serum neutralization test, electron microscope observation, or the molecular detection of nucleic acid (RT-PCR, including real-time RT-PCR). The cell culture of NiV is less affected by virus variations than other methods and the virus features can be described in detail (Hyatt et al., 2001). The serum neutralization test is the standard test method of detecting NiV (Peter et al., 2001). However, both of these methods require BSL-4 facilities. The detection of NiV with an ELISA is reliable (Yadav et al., 2012), but it’s less sensitive than its molecular detection. Electron microscopy is unable to distinguish NiV from Hev or other paramyxoviruses (Chow et al., 2000). RT-PCR and Real-time RT-PCR are sensitive methods for the rapid detection of NiV (Guillaume et al., 2004). In this study, we established an RT-LAMP assay for NiV all known strains detection, which may offer an additional molecular technique for rapid, specific and sensitive identification of NiV. The RT-LAMP assay targets a conserved region of the N gene, determined with a multiple sequence alignment of the NiV N genes. The six RT-LAMP primers (F3, B3, FIP, BIP, LF, and LB) were designed to target this region. All of the experiments were performed in triplicate. The RT-LAMP primers recognized all the known NiV strains, and the results were available within 45 min with the Realtime Turbidimeter and within 50 min when detected with 1 μl of calcein indicator in a water bath, whereas other molecular detection methods take at least 1–2 h. This RT-LAMP assay is at least 10 times more sensitive than conventional RT-PCR, and can detect approximately 100 pg of total NiV pseudovirus RNA. More importantly, the RT-LAMP assay shows good stability, which was demonstrated with clinical samples, so the impurities in clinical samples had no or little effect on the sensitivity of the assay, and the assay is suitable for clinical use. Guillaume et al. reported a real-time RT-PCR (TaqMan) assay for NiV in 2004 (Guillaume et al., 2004) that also targeted the N gene, and the sensitivity of the assay was close to 1 pfu. Analogously, Foord et al. established a LAMP method for the rapid detection of HeV (Foord et al., 2012), with the same sensitive with TaqMan assay. However, if we improve the purity of the RT-LAMP primers with High Performance Liquid Chromatography (HPLC), the sensitivity of the assay may increase, as reported by Norihiro et al. (2008), but this proposition requires verification.

From the evidences presented in this study, the assay we have developed may offer an additional molecular detection method for the rapid, specific and sensitive identification of NiV, although further optimization and verification are required. Notably, this RT-LAMP assay can detect NiV under an isothermal conditions, using only a thermostatically controlled water bath or metal bath, and the results can be read with the naked eye after the addition of 1 μl of calcein indicator. The RT-LAMP assay can also be applied for routine epidemiological surveillance and the detection of disease outbreaks.

## Author Contributions

DL and QC mainly supervised the study. LM designed and conducted the experiments, analyzed the data, and drafted the manuscript. ZC and WG designed and constructed the pseudoviruses of the NiV N gene.

## Conflict of Interest Statement

The authors declare that the research was conducted in the absence of any commercial or financial relationships that could be construed as a potential conflict of interest.

## Footnotes

1 http://primerexplorer.jp/lampv5e/index.html
